# The interrelationship and accumulation of cardiometabolic risk factors amongst young adults in the United Arab Emirates: The UAE Healthy Future Study

**DOI:** 10.1186/s13098-021-00758-w

**Published:** 2021-11-27

**Authors:** Fatima Mezhal, Abderrahim Oulhaj, Abdishakur Abdulle, Abdulla AlJunaibi, Abdulla Alnaeemi, Amar Ahmad, Andrea Leinberger-Jabari, Ayesha S. Al Dhaheri, E. Murat Tuzcu, Eiman AlZaabi, Fatma Al-Maskari, Fatme Alanouti, Fayza Alameri, Habiba Alsafar, Hamad Alblooshi, Juma Alkaabi, Laila Abdel Wareth, Mai Aljaber, Marina Kazim, Micheal Weitzman, Mohammad Al-Houqani, Mohammad Hag Ali, Naima Oumeziane, Omar El-Shahawy, Rami H. Al-Rifai, Scott Scherman, Syed M. Shah, Tom Loney, Wael Almahmeed, Youssef Idaghdour, Luai A. Ahmed, Raghib Ali

**Affiliations:** 1 Public Health Research Center, New York University Abu Dhabi, Abu Dhabi, UAE; 2 Department of Epidemiology and Public Health, College of Medicine and Health Sciences, Khalifa University of Sciences and Technology, Abu Dhabi, UAE; 3 Department of Pediatrics, Zayed Military Hospital, Abu Dhabi, UAE; 4 Department of Cardiology, Zayed Military Hospital, Abu Dhabi, UAE; 5 Department of Nutrition and Health, College of Medicine and Health Sciences, United Arab Emirates University, Al-Ain, UAE; 6 Heart and Vascular Institute, Cleveland Clinic Abu Dhabi, Abu Dhabi, UAE; 7 Department of Pathology, Sheikh Shakhbout Medical City, Abu Dhabi, UAE; 8 Institute of Public Health, College of Medicine and Health Sciences, United Arab Emirates University, Al-Ain, UAE; 9 Zayed Center for Health Sciences, United Arab Emirates University, Al-Ain, UAE; 10 College of Natural and Health Sciences, Zayed University, Abu Dhabi, UAE; 11 Zayed Military Hospital, Abu Dhabi, UAE; 12 Center for Biotechnology, Khalifa University of Science and Technology, Abu Dhabi, UAE; 13 Department of Genetics and Molecular Biology, Khalifa University of Science and Technology, Abu Dhabi, UAE; 14 Department of Biomedical Engineering, Khalifa University of Science and Technology, Abu Dhabi, UAE; 15 Abu Dhabi Blood Bank Services, SEHA, Al-Ain, Abu Dhabi UAE; 16 Department of Internal Medicine, College of Medicine and Health Sciences, United Arab Emirates University, Al-Ain, UAE; 17 Pathology and Laboratory Medicine Institute, Cleveland Clinic Abu Dhabi, Abu Dhabi, UAE; 18 Healthpoint Hospital, Abu Dhabi, UAE; 19 Department of Environmental Medicine, New York University of Medicine, New York, USA; 20 Department of Medicine, College of Medicine and Health Sciences, United Arab Emirates University, Al-Ain, UAE; 21 Department of Health Science, Higher Colleges of Technology, Abu Dhabi, UAE; 22 Department of Population Health, New York University School of Medicine, New York, USA; 23 College of Medicine, Mohammed Bin Rashid University of Medicine and Health Sciences, Dubai, UAE; 24 MRC Epidemiology Unit, University of Cambridge, Cambridge, UK

**Keywords:** Cardiovascular disease, Cardiometabolic risk factors, Obesity, Dysglycemia, Dyslipidemia, Hypertension, Central obesity, Metabolic Syndrome

## Abstract

**Introduction:**

Similar to other non-communicable diseases (NCDs), people who develop cardiovascular disease (CVD) typically have more than one risk factor. The clustering of cardiovascular risk factors begins in youth, early adulthood, and middle age. The presence of multiple risk factors simultaneously has been shown to increase the risk for atherosclerosis development in young and middle-aged adults and risk of CVD in middle age.

**Objective:**

This study aimed to address the interrelationship of CVD risk factors and their accumulation in a large sample of young adults in the United Arab Emirates (UAE).

**Methods:**

Baseline data was drawn from the UAE Healthy Future Study (UAEHFS), a volunteer-based multicenter study that recruits Emirati nationals. Data of participants aged 18 to 40 years was used for cross-sectional analysis. Demographic and health information was collected through self-reported questionnaires. Anthropometric data and blood pressure were measured, and blood samples were collected.

**Results:**

A total of 5126 participants were included in the analysis. Comorbidity analyses showed that dyslipidemia and obesity co-existed with other cardiometabolic risk factors (CRFs) more than 70% and 50% of the time, respectively. Multivariate logistic regression analysis of the risk factors with age and gender showed that all risk factors were highly associated with each other. The strongest relationship was found with obesity; it was associated with four-fold increase in the odds of having central obesity [adjusted OR 4.70 (95% CI (4.04–5.46)], and almost three-fold increase odds of having abnormal glycemic status [AOR 2.98 (95% (CI 2.49–3.55))], hypertension (AOR 3.03 (95% CI (2.61–3.52))] and dyslipidemia [AOR 2.71 (95% CI (2.32–3.15)]. Forty percent of the population accumulated more than 2 risk factors, and the burden increased with age.

**Conclusion:**

In this young population, cardiometabolic risk factors are highly prevalent and are associated with each other, therefore creating a heavy burden of risk factors. This forecasts an increase in the burden of CVD in the UAE. The robust longitudinal design of the UAEHFS will enable researchers to understand how risk factors cluster before disease develops. This knowledge will offer a novel approach to design group-specific preventive measures for CVD development.

## Introduction

Cardiovascular disease (CVD) remains the number one cause of death and disability in the world. About 85% of CVD deaths are attributable to ischemic heart disease and stroke [1]. There are multiple risk factors associated with CVD. The most common ones include general obesity (based on BMI), central obesity or abdominal obesity, hyperglycemia, dyslipidemia, and high-blood pressure. The prevalence of the cardiometabolic risk factors (CRFs) associated with non-communicable diseases (NCDs) has increased in the UAE and will continue to increase, as demonstrated by many studies and as predicted by projections and future estimates [2].

Having one risk factor does not necessarily lead to developing CVD. Similar to other NCDs, people who develop ischemic heart disease typically have more than one risk factor. The clustering of cardiovascular risk factors begins in youth, and continues during young adulthood and middle age [3, 4]. The presence of multiple risk factors simultaneously has been shown to increase the risk for atherosclerosis development in young and middle-aged adults and risk of CVD in middle age [5].

For example, Wilson et al.’s [6]study estimated that accumulating three or more risk factors was associated with around a 2.4-fold increase in men and 5.9-fold increase in women in the risk of coronary heart disease after 16 years of follow-up. Additionally, they showed that having 3 or more risk factors in the general population, was attributable to about 20% of coronary events in men and 48% in women. Another study on hypertensive individuals without CVD, showed that accumulating three or more risk factors increased the relative risk of developing cardiovascular events from 2.07 (95% CI 1.86–2.30) to 2.80 (95% CI 2.48–3.17) when compared to having only one risk factor, in a 6-year follow up [7].

Interrelationships between pairs of risk factors have been studied previously. Weight increase was reported to be associated with hyperlipidemia, glycaemia, and hypertension in young adults [8]. Hypertension was reported to be associated with type 2 diabetes [9]. In addition, insulin resistance was associated with hypertension [10]. Other studies reported an increase in incident diabetes and hypertension following dyslipidemia [11, 12].

Since NCDs are caused by the interplay of risk factors and their accumulation, it is important to study how these risk factors are linked and how they accumulate before a chronic disease is established. The majority of local research in UAE has studied the risk factors individually. Most of the epidemiological studies are pre-dated and recruited a sample from a particular geographic location (e.g. city) or from specific healthcare settings. Although it is well-established that chronic diseases start developing in younger adults, there are limited studies in the UAE on the burden of risk factors in young adults. This study aimed to address the accumulation of cardiometabolic risk factors (CRFs) and their interrelationship in a large sample of adults below 40 years.

## Methods

### Study sample

The study participants were from the UAE Healthy Future Study (UAEHFS) [13]. The UAEHFS is an ongoing population-based prospective cohort study that aims to explore risk factors for NCDs. Emirati adults are invited to participate at multiple centers across major cities in the UAE.The study was based on the cross-sectional analysis of available baseline data from the UAEHFS cohort, recruited between February 2016 and December 2018. Subjects were nationals aged 18 to 40 years. All participants provided informed consent. Participants who reported any acute infection at the time of recruitment and pregnant women were excluded from the study. This study was approved by the Abu Dhabi Health Research and Technology Committee (ref. DOH/HQD/2020/516). Additional information on the UAEHFS methodology is published elsewhere [13].

### Data collection

Participants answered a self-completed questionnaire that collected socio-demographic, health, and lifestyle information. Participants underwent physical measurements including height, weight, waist and hip circumferences as well as blood pressure measurements. Blood samples were collected to measure glycated hemoglobin (HbA1c), low-density lipoprotein (LDL) cholesterol, high density lipoprotein (HDL) cholesterol, total cholesterol, and triglycerides. Only fasting samples were used to measure blood glucose.

### Cardiometabolic risk factors criteria

Body mass index (BMI) was categorized according to the WHO definitions. A BMI less than 25.0 kg/m^2^ was considered normal. A BMI between 25.0 and 29.9 kg/m^2^ was considered overweight. And a BMI ≥ 30.0 kg/m^2^ was classified as obese.

Dysglycemia, or abnormal glycemic status, was defined as prediabetes or diabetes. Cut-offs were set at HbA1c ≥ 5.7% and < 6.5%, and fasting blood glucose (FBG) ≥ 100 and < 126 mg/dL for prediabetes, while diabetes was classified as having HbA1c ≥ 6.5%, FBG ≥ 126 mg/dl and/or reporting diabetes or taking antidiabetic medication [14, 15].

Dyslipidemia was defined as either self-reported history of abnormal cholesterol level, or taking a lipid-controlling medication or having an abnormal level of any of the following; LDL cholesterol level of ≥ 130 mg/dL, HDL cholesterol level of ≤ 40 mg/dL for men or ≤ 50 mg/dL for women, total cholesterol ≥ 200 mg/dL or triglycerides ≥ 150 mg/dL for fasting samples and ≥ 175 mg/dL for random samples [16, 17].

Elevated blood pressure, or hypertension, was defined as having two consecutive blood pressure readings of ≥ 140 mmHg systolic and/or ≥ 90 mmHg diastolic according to the American Heart Association guidelines [18]. Hypertension was also defined as having self-reported “hypertension” on the questionnaire and/or whether they are taking blood pressure-controlling medication.

Abdominal obesity (or central obesity) was indicated if the waist-to-hip ratio ≥ 0.85 for women and ≥ 0.90 for men [19].

### Statistical analyses

Baseline characteristics of the study participants were presented overall and by gender. Categorical data was presented as frequencies and percentages and continuous variables were presented as means ± standard deviation. The frequencies and percentages were tested for significance of any differences in distribution between two or more groups using chi-square test. For continuous variables, differences in means were measured by Welch t-tests. The prevalence was adjusted for age using logistic models and presented with 95% confidence intervals (CI).

The burden of CRFs was defined and estimated as the number of risk factors per individual. The maximum number of risk factors was five per individual. CRFs were then grouped to form two burden groups; “0–1” and “ ≥ 2” risk factors. Multivariate logistic regression analyses were performed to measure the associations between CRFs, adjusted for age and gender. Adjusted Odds Ratios (OR) with their 95% CIs were reported. The analyses were performed using Stata 15 software [20]. The significance level of the statistical tests was set at 5%.

## Results

A total of 5167 subjects aged between of 18 and 40 years were recruited from February 2016 to December 2018. Questionnaire data was available for up to 85% of the participants, anthropometric data and blood pressure was available for 94% of the sample, and blood biomarkers data was available for 98% of the sample. More than 80% of the population had complete data points. Table 1 represents the age-adjusted cardiometabolic characteristics of the study population.

**Table 1:** Age-adjusted prevalence % of cardiometabolic risk factors of UAEHFS participants

	Overall N = 5126	Men, *N* = 3202 (62%)	Women, *N* = 1965 (38%)	P-value
Age (years), mean (SD)	25.7 (6.2)	26.4 (5.9)	24.5 (6.3)	< 0.001
Overweight	30.1 (28.8–31.4)	34.7 (33.0–36.4)	23.1 (21.2–25.0)	< 0.001
Obesity	26.5 (25.2–27.7)	29.7 (28.0–31.4)	21.6 (19.7–23.5)	< 0.001
Prediabetes	8.2 (7.4–8.9)	10.1 (9.1–11.2)	5.2 (4.1–6.2)	< 0.001
Diabetes	3.5 (3.0–4.0)	3.8 (3.1–4.5)	3.1 (2.3–3.9)	< 0.001
Dysglycemia	11.7 (10.8–12.7)	14 (12.7–15.2)	8.3 (7.0–9.6)	< 0.001
High LDL	34.5 (33.2–35.9)	42.1 (40.3–43.9)	22.9 (20.9–24.8)	< 0.001
Low HDL	43.7 (42.4–45.1)	45.4 (43.6–47.1)	41.1 (38.9–43.3)	0.003
High total cholesterol	32.8 (31.4–34.1)	37.2 (35.4–38.9)	26.0 (24.0–28)	< 0.001
High Triglycerides	21.4 (20.2–22.6)	26.7 (25.1–28.3)	13.5 (11.9–15.1)	< 0.001
Dyslipidemia	62.7 (61.3–64)	68 (66.3–69.7)	54.2 (52–56.5)	< 0.001
Hypertension	22.4 (21.2–23.6)	30.9 (29.2–32.6)	9.2 (7.8–10.5)	< 0.001
Central obesity	22.5 (21.3–23.8)	29.6 (27.9–31.3)	12.5 (10.9–14.0)	< 0.001

Age is presented as mean years (standard deviation). Data is presented as prevalence % (confidence interval)

Overweight and obesity were defined as having a BMI between 25.0 and 29.9 kg/m^2^ and BMI ≥ 30 kg/m^2^, respectively. Dysglycemia was defined as having HbA1c ≥ 5.7%, and/or FBG ≥ 100 mg/dl, and/or self-reporting diabetes or taking antidiabetic medication. Dyslipidemia was defined as having any abnormality across lipid markers (LDL ≥ 130 mg/dl, HDL ≤ 40 mg/dl for men or ≤ 50 mg/dl for women, total cholesterol ≥ 200 mg/dl or triglycerides ≥ 150 mg/dl for fasting samples and ≥ 175 mg/dl for random samples) and/or self-reporting abnormal cholesterol or taking lipid-controlling medication. Hypertension was defined as ≥ 140 mmhg systolic and/or ≥ 90 mmhg diastolic pressure and/or self-reporting hypertension or taking blood pressure-controlling medication. Central obesity was defined as having waist-to-hip ratio as ≥ 0.85 for women and ≥ 0.90 for men

Almost two-thirds of the sample was classified as either overweight or obese, 30.1% [95% CI(28.8−31.4)] and 26.5% [95% CI (25.2–27.7)]; respectively. Men had higher prevalence than women (p < 0.001). Both prediabetes and diabetes prevalence were estimated as 8.2% [95%CI (7.4–8.9)]and 3.5% [95%CI (3.0–4.0)]; respectively. Abnormal glycemic markers were higher in men than women (p < 0.001). Moreover, abnormal lipid biomarkers were consistently higher in men than women (p < 0.001), contributing to a total dyslipidemia prevalence of 68.0% [95% CI (66.3–69.7)] in men and 54.2% [95% CI (52.0–56.5)] in women (p < 0.001).

Hypertension, based on blood pressure measurements and self-report, was estimated as 22.4% [95%CI (21.2–23.6)]; significantly higher in men than women; 30.9% [95%CI (29.2–32.6)] and 9.2% [95%CI (7.8–10.5)] (*P* < 0.001); respectively. Finally, abdominal obesity was estimated as 22.5% [95%CI (21.3–23.8)] in the whole sample and the prevalence was more than double in men compared to women (P < 0.001).

Table 2 presents a summary of the prevalence of cardiometabolic comorbidity; having two cardiovascular risk factors simultaneously. Among the people with dyslipidemia, more than 70% had another coexisting metabolic risk factor. The following most common co-existing risk factor was obesity. More than 50% of obese participants also have had dysglycemia or central obesity. Interestingly, among dysglycemic participants, only 24% were also classified as obese, and 23% classified as hypertensive.

**Table 2:** The prevalence of comorbidity of cardiometabolic risk factors in the UAEHFS participants

	Obesity	Dysglycemia	Dyslipidemia	Hypertension	Central obesity
Central obesity	48.9 (46.2–51.6)	37.9 (34.1–41.8)	31.1 (29.5–32.8)	38.9 (36–41.8)	
Hypertension	39.4 (36.8–42.1)	40.8 (37–44.7)	27.8 (26.3–29.5)		36.6 (33.8–39.4)
Dyslipidemia	79.4 (77.2–81.5)	77.3 (73.9–80.4)		75.2 (72.6–77.6)	79.1 (76.6–81.4)
Dysglycemia	24 (21.8–26.4)		15.7 (14.5–17)	22.8 (20.5–25.4)	19.7 (17.6–22.1)
Obesity		51.7 (47.8–55.6)	35.1 (33.3–36.8)	47.4 (44.4–50.4)	54.9 (52–57.8)

Data is presented as prevalence % (95% CI) for co-existing CRFs

Associations among the risk factors were investigated. Table 3 presents the associations between the five CRFs adjusting for age and gender. The strongest relationship was captured with obesity. For instance, obesity was associated with more than four-fold increase in the odds of having central obesity [OR 4.70 95%CI (4.04–5.46)], and almost three-fold increase in the odds of having abnormal glycemic status [OR 2.98 95%CI (2.49–3.55)], hypertension [OR 3.03 95%CI (2.61–3.52)], and dyslipidemia [OR 2.71 95% CI (2.32–3.15)].

**Table 3:** Odd ratios of the associations between the cardiometabolic risk factors adjusted for age and sex

	Obesity	Dysglycemia	Dyslipidemia	Hypertension
Central obesity	4.70 (4.04–5.46)	1.57 (1.29–1.9)	2.18 (1.85–2.56)	1.85 (1.58–2.17)
Hypertension	3.03 (2.61–3.52)	2.32 (1.92–2.79)	1.81 (1.54–2.12)	
Dyslipidemia	2.71 (2.32–3.15)	1.85 (1.51–2.26)		
Dysglycemia	2.98 (2.49–3.55)			

Data is presented as odds ratios (95% CI). Multivariate models adjusted for age and gender only. For each risk factor, the reference groups were those without that risk factor

**Fig. 1:**
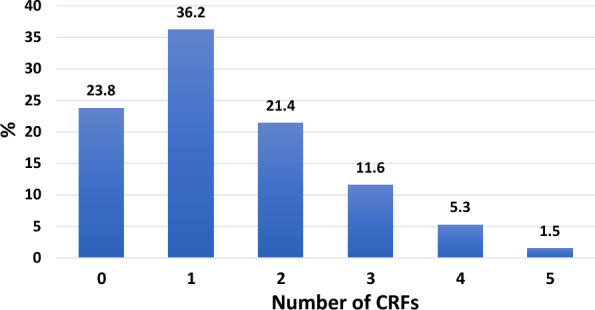
Age-adjusted prevalence of number of accumulated cardiometabolic risk factors in the whole sample

**Fig. 2:**
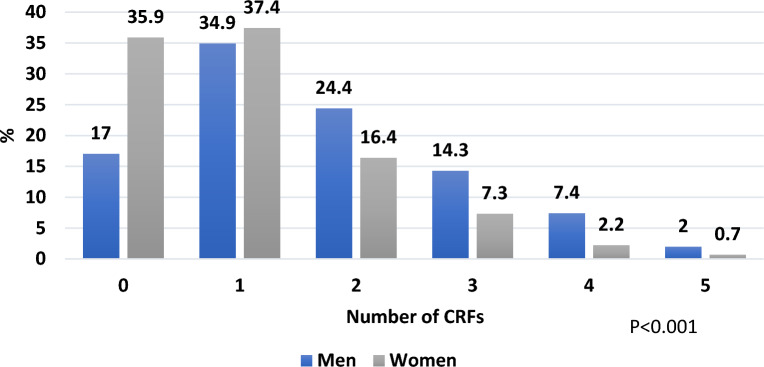
Age- adjusted prevalence of accumulated cardiometabolic risk factors in men and women

The burden of CRFs was measured as the number of risk factors accumulated per subject. Around a quarter of the population (23.8%) had zero risk factors. The remaining population had a range from 1 to 5 risk factors. The majority of the sample had either 1 risk factor (36.2%) or 2 risk factors (21.4%) as displayed in Fig. 1. The distribution of number of risk factors in men and women is visualized in Fig. 2. Males in this sample had more risk factors than females; 83% of men had at least one risk factor versus 64% of women (p < 0.001).

**Fig. 3:**
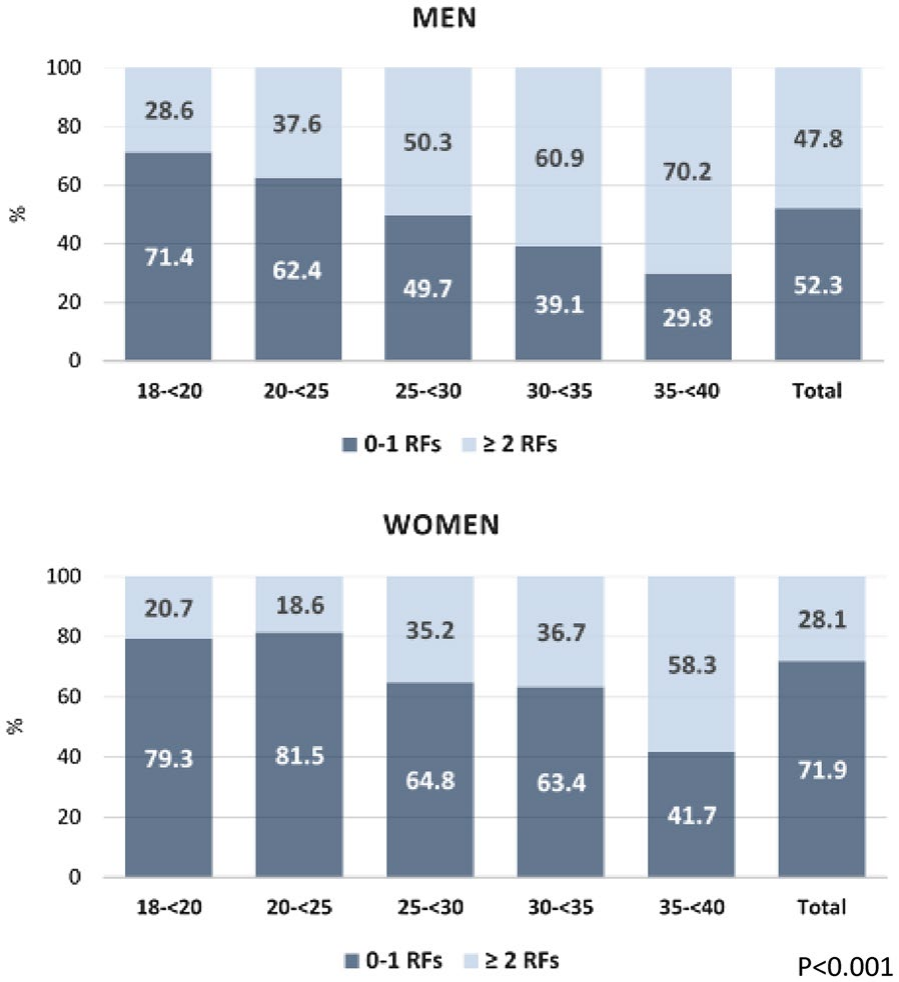
Burden of cardiometabolic risk factors in different age groups

Figure 3 shows the distribution of the burden of the CRFs dichotomized to “0–1 RFs” and “ ≥ 2 RFs” within age groups. About sixty percent of the total population had 0–1 risk factors.The proportion of accumulated risk factors increased in the older age groups (p < 0.001). This was similar in men and women.

**Fig. 4:**
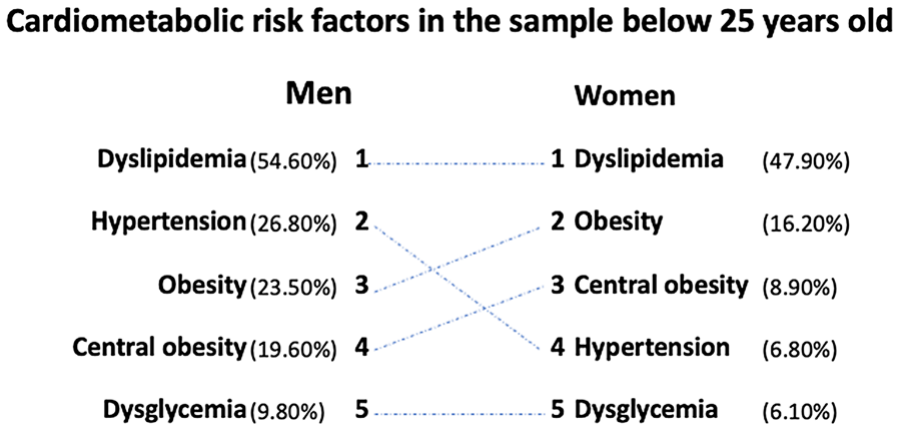
Prevalence of risk factors in UAEHFS below the age of 25 years in men and women

Figure 4 represents the most common CRFs in the youngest age groups, those below the age of 25 years. In men, the most common CRF was dyslipidemia, where it was reported in 54.6% of the male population, followed by hypertension, obesity, central obesity then dysglycemia. In women, however, the rankings differed. The most common CRF was dyslipidemia, followed by obesity, central obesity, hypertension then dysglycemia.

## Discussion

This study presents the first comprehensive description of the accumulation of common CVD risk factors and their interrelationship in a large sample of young Emirati adults. It is well established that before CVD develops, multiple risk factors co-exist. The clustering of the cardiovascular risk factors starts in adolescence and early adulthood [4]. In this study, we investigated the extent of co-existence of these risk factors and how often do they accumulate in young adults between 18 and 40 years.

The study showed interesting patterns in this population. We found that dyslipidemia coexisted with another metabolic abnormality more than 75% of the time, followed by obesity and central obesity. Interestingly, in the dysglycemic group, comorbidity was least evident. In contrast, in another national study, Hajat et al. showed that cardiometabolic comorbidity was most evident in diabetic participants [21].

All associations were found to be significant after adjusting for age and gender (Table 3). The associations between pairs of CRFs indicate that these risk factors cluster differently in people. Overall, obesity had the strongest relationship with all metabolic abnormalities. Baynouna et al. [22] showed that the strongest interrelationship between risk factors was detected with obesity and hypertension, with an odd ratio 1.9 (95%CI 1.2 – 3.0), and with high LDL, odd ratio 1.7 (95%CI 1.1–2.5).

One quarter of the total sample population had no CRFs. Grouping the burden into two categories, 0–1 and 2 or more risk factors yielded a 60–40 ratio; 60% had one or no risk factor, and 40 had two or more risk factors. Almost half (47.8%) of the male population in this study had two or more risk factors, while only 28.1% of the female’s population did. This should be considered as alarming as this is a young population sample with aged between 18–40 years. Surprisingly, even 24% of the subjects in the youngest age group 18–19 years had already developed 2 or more CRFs.

Focusing more on the youngest age groups (below 25 years), we have found that CRFs prevalence differ across men and women. Figure 4 shows that in men, dyslipidemia and hypertension are the highest 2 CRFs among young men, while obesity ranks third. In young women, hypertension rates were much lower and ranked fourth, after dyslipidemia, obesity and central adiposity. The finding that dyslipidemia has the highest prevalence and also almost always co-existing with other CRFs is in line with Paynter et al.‘s finding [5]. They have reported that dyslipidemia is more likely to occur first in a cluster of risk factors, more than hypertension, obesity and diabetes. These findings strongly suggest that dyslipidemia screening in early adulthood may be a good target for risk factor accumulation and therefore CVD prevention.

A recent report from the UAE National Health Survey estimated that 49.5% of the 18–44 years’ population had three or more of the following risk factors: smoking, inadequate diet, insufficient physical activity, overweight, or raised blood pressure [23]. The survey results indicated that there were more men than women with such criteria (54.4 vs. 45.1% respectively). However, this estimation was not limited to Emiratis and included burden of non-metabolic risk factors. In another report on the burden of cardiovascular risk factors in 33,000 young military men, it was estimated that 24% had at least 2 risk factors [24]. This prevalence probably underestimates the burden at population level as the sample subjects were military men, which have differences in age structure, social and behavioral characteristics, and health and physical fitness standards required for their occupation.

We also found that the proportion of people having 2 or more risk factors increased with age. This finding parallels the established fact that cardiovascular risk increases with age. It is well known that aging increases the risk for CVD as there are multiple structural and functional alterations that occur throughout a lifespan [25]. For instance, changes at the molecular level, such as the increase in oxidative stress can lead to obesity, diabetes, and frailty, which is called “cardiovascular aging” [26].

The main strength of this study is the ability to asses CVD risk factors in young adults in large population-based sample size. This study focused on young adults, who are often underrepresented in the context of non-communicable disease studies, especially CVD. Cardiovascular risk factors definitions were thorough and included objective and subjective measures for a more concise disease-definition criteria. Blood samples and measurements were collected in a standardized procedure to ensure consistent quality and reduce the risk of information bias.

In this study, we defined dyslipidemia as having any abnormality across the 4 lipids biomarkers: LDL, HDL, total cholesterol, and triglycerides, as well as reporting a medical diagnosis and/or taking medication. This definition is recommended by the ATP 3 guidelines for persons above 20 years old [27]. Besides the broad definition, we used random non-fasting samples, which recent reports have shown are equally acceptable [28, 29].

Most epidemiological studies are prone to having selection bias that can affect the external validity of the study. The main weakness of this study is that it is volunteer-based recruitment of participants, which therefore potentially affects the representativeness of the study sample. However this is common to most large cohort studies which are volunteer-based convenience samples, but provided that there is wide range of exposures within the cohort, this is not a significant limitation to understanding the relationship between exposures and outcomes.

## Conclusion

Studying the major cardiovascular risk factors and how they link and accumulate to each other in a young sample of Emiratis provides a novel insight. Here, we showed how the major risk factors are highly prevalent and start accumulating very early in age, even in those below 25 years of age. The population’s increasing burden of risk factors forecasts an increase in the future incidence of CVD. This calls for taking preventive measures that must be designed for the youth in schools and universities. Also, comorbidity analysis in this study showed that dyslipidemia co-exists with other cardiometabolic abnormalities. Such patients must be additionally screened for other risk factors and must be made aware that they would be more prone for having another metabolic abnormality.

Finally, addressing the high burden of risk factors is only a first step in understanding how clustering will affect the incidence of CVD. Studying the basis for risk factor clustering will provide insight into the pathogenesis of atherosclerosis and it has implications for the prevention of coronary disease.

## Data Availability

Data is available upon request.

## Abbreviations

NCDs: Non-communicable disease
CVD: Cardiovascular disease
UAE: United Arab Emirates
UAEHFS: UAE Healthy Future Study
CRFs: Cardiometabolic risk factors
AOR: Adjusted odds ratio
OR: Odds ratio
CI: Confidence interval
HbA1c: Hemoglobin A1c, glycated hemoglobin
LDL: Low-desity lipoprotein
HDL: High-density lipoprotein
BMI: Body mass index
WHO: World Health Organization
FBG: Fasting blood glucose
SD: Standard deviation
