# Correction: The interrelationship and accumulation of cardiometabolic risk factors amongst young adults in the United Arab Emirates: The UAE Healthy Future Study

**DOI:** 10.1186/s13098-024-01279-y

**Published:** 2024-02-07

**Authors:** Fatima Mezhal, Abderrahim Oulhaj, Abdishakur Abdulle, Abdulla AlJunaibi, Abdulla Alnaeemi, Amar Ahmad, Andrea Leinberger‑Jabari, Ayesha S. Al Dhaheri, E. Murat Tuzcu, Eiman AlZaabi, Fatma Al‑Maskari, Fatme Alanouti, Fayza Alameri, Habiba Alsafar, Hamad Alblooshi, Juma Alkaabi, Laila Abdel Wareth, Mai Aljaber, Marina Kazim, Micheal Weitzman, Mohammad Al‑Houqani, Mohammad Hag Ali, Naima Oumeziane, Omar El‑Shahawy, Rami H. Al‑Rifai, Scott Scherman, Syed M. Shah, Tom Loney, Wael Almahmeed, Youssef Idaghdour, Luai A. Ahmed, Raghib Ali

**Affiliations:** 1 Public Health Research Center, New York University Abu Dhabi, Abu Dhabi, UAE; 2 Department of Epidemiology and Public Health, College of Medicine and Health Sciences, Khalifa University of Sciences and Technology, Abu Dhabi, UAE; 3 Department of Pediatrics, Zayed Military Hospital, Abu Dhabi, UAE; 4 Department of Cardiology, Zayed Military Hospital, Abu Dhabi, UAE; 5 Department of Nutrition and Health, College of Medicine and Health Sciences, United Arab Emirates University, Al‑Ain, UAE; 6 Heart and Vascular Institute, Cleveland Clinic Abu Dhabi, Abu Dhabi, UAE; 7 Department of Pathology, Sheikh Shakhbout Medical City, Abu Dhabi, UAE; 8 Institute of Public Health, College of Medicine and Health Sciences, United Arab Emirates University, Al‑Ain, UAE; 9 Zayed Center for Health Sciences, United Arab Emirates University, Al‑Ain, UAE; 10 College of Natural and Health Sciences, Zayed University, Abu Dhabi, UAE; 11 Zayed Military Hospital, Abu Dhabi, UAE; 12 Center for Biotechnology, Khalifa University of Science and Technology, Abu Dhabi, UAE; 13 Department of Genetics and Molecular Biology, Khalifa University of Science and Technology, Abu Dhabi, UAE; 14 Department of Biomedical Engineering, Khalifa University of Science and Technology, Abu Dhabi, UAE; 15 Abu Dhabi Blood Bank Services, SEHA, Al‑Ain, Abu Dhabi UAE; 16 Department of Internal Medicine, College of Medicine and Health Sciences, United Arab Emirates University, Al‑Ain, UAE; 17 Pathology and Laboratory Medicine Institute, Cleveland Clinic Abu Dhabi, Abu Dhabi, UAE; 18 Healthpoint Hospital, Abu Dhabi, UAE; 19 Department of Environmental Medicine, New York University of Medicine, New York, USA; 20 Department of Medicine, College of Medicine and Health Sciences, United Arab Emirates University, Al‑Ain, UAE; 21 Department of Health Science, Higher Colleges of Technology, Abu Dhabi, UAE; 22 Department of Population Health, New York University School of Medicine, New York, USA; 23 College of Medicine, Mohammed Bin Rashid University of Medicine and Health Sciences, Dubai, UAE; 24 MRC Epidemiology Unit, University of Cambridge, Cambridge, UK

**Correction: Diabetology & Metabolic Syndrome (2021) 13:140** 10.1186/s13098-021-00758-w

Following publication of the original article [1], the authors identified an error in Table 3. The word “Dyslipidemia” should be removed from Hypertension column.

The correct Table 3 is given below.

**Table 3:** Odd ratios of the associations between the cardiometabolic risk factors adjusted for age and sex

	Obesity	Dysglycemia	Dyslipidemia	Hypertension
Central obesity	4.70 (4.04–5.46)	1.57 (1.29–1.9)	2.18 (1.85–2.56)	1.85 (1.58–2.17)
Hypertension	3.03 (2.61–3.52)	2.32 (1.92–2.79)	1.81 (1.54–2.12)	
Dyslipidemia	2.71 (2.32–3.15)	1.85 (1.51–2.26)		
Dysglycemia	2.98 (2.49–3.55)			

Data is presented as odds ratios (95% CI). Multivariate models adjusted for age and gender only. For each risk factor, the reference groups were those without that risk factor

The original article [1] has been revised.
